# On Extra-Uterine Pregnancy

**Published:** 1814-12

**Authors:** M. Chausier


*For the Medical and Physical Journal.*

### *On Extra-Uterine Pregnancy;* by M. CHAUSIER.

A WOMAN, thirty years of age, who enjoyed a good state of health, and had been three times pregnant, conceived herself to be in the same situation in the month of December. She felt, however, more weight than in former pregnancies, and a sense of uneasiness on the left side, to which she had never been accustomed. The gradual enlargement of the belly, the swelling of the mammæ, and es-

---

pecially an inward movement, which became evident between the third and fourth month, left no doubt of her being with child; but at this period she complained of a fixed tensive and occasionally lancinating pain from the left ilium to the kidney on the same side, with scarcely any interval of ease. The nights now became troubled, the sleep disturbed, the appetite lost, and the strength diminished. At the fifth month, the motion of the child ceased, but the pains were become more acute and constant, extending from the lower part of the abdomen to the region of the umbilicus. Fever now supervened, with excessive thirst, sleeplessness, total loss of appetite, and sometimes diarrhœa. On the 30th of May she was received into the Hospital Maternité, then calculating it was the sixth month of her pregnancy. Her countenance was cadaverous; eyes hollow; the emaciation was extreme; respiration short, difficult, and interrupted; pulse weak, feeble, and depressed. The tension and sensibility of the abdomen were so great, that the least pressure could not be borne.* Finally, the labia and the left thigh were œdematous. On examination per vaginam, it was discovered that *the neck of the uterus preserved its natural length*: it was soft, thick, and its external orifice was *open*. On the left side, at the posterior part of the vagina, a projection was felt, seemingly of a tumour deeply situated. On the following morning, as the pains increased, she was examined again. The *mouth of the uterus was entirely open*, and gave issue to a sanguineo-mucous discharge. The finger could be readily passed into the uterus, whose parietes were thickened and cavity enlarged, but it was evidently empty. Taking this into consideration, and the tumour felt on the left side of the vagina, left no reason to doubt that the symptoms arose from a fœtus contained in the left fallopian tube. Thirty-eight hours after her admission into the hospital she died.

*Dissection.*—Water was found in the chest and pericardium. On opening the abdomen, a black fœtid fluid escaped. The stomach, liver, and spleen were healthy, but the omentum and intestines, which adhered together at every point of contact, were in a gangrenous state, concealing a large, soft,

* Why this patient was not bled will be a matter of astonishment to all who are unacquainted with the negligence of the French physicians in this particular. It might have relieved the symptoms, though no hope remained of the recovery of the patient unless by an incision into the abdomen for the purpose of extracting the fœtus.—TRANSLATOR.

and and oval tumour arising from the left side of the pelvis, and occupying the left hypogastric region.

Endeavouring to raise the intestinal mass, and to detach it from the adhesions it had formed with different portions of the tumour, the latter was ruptured at its anterior part, and a fœtus escaped, with a large quantity of yellowish fluid. The cyst containing the fœtus proved to be the left fallopian tube. The walls were thin, but very vascular. The placenta attached to the inner surface of the cyst was broad and thin. When detached, the *membrana caduca* of Dr. Hunter was very distinct, and the same membrane was discovered in the uterus itself.—*Journ. de Med. May*, 1814.

